# The national health insurance scheme: perceptions and experiences of health care providers and clients in two districts of Ghana

**DOI:** 10.1186/2191-1991-2-13

**Published:** 2012-07-23

**Authors:** Philip Ayizem Dalinjong, Alexander Suuk Laar

**Affiliations:** 1 Navrongo Health Research Centre, Post Office Box 114, Navrongo, Upper East Region, Ghana; 2 National Health Insurance Authority (Operations), Greater Accra Region, Accra, Ghana

**Keywords:** National health insurance, Social health insurance, Universal health coverage, Perceptions, Experiences, Health care providers’ behavior, Insured, Uninsured, Ghana

## Abstract

**Background:**

Prepayments and risk pooling through social health insurance has been advocated by international development organizations. Social health insurance is seen as a mechanism that helps mobilize resources for health, pool risk, and provide more access to health care services for the poor. Hence Ghana implemented the National Health Insurance Scheme (NHIS) to help promote access to health care services for Ghanaians. The study examined the influence of the NHIS on the behavior of health care providers in their treatment of insured and uninsured clients.

**Methods:**

The study took place in Bolgatanga (urban) and Builsa (rural) districts in Ghana. Data was collected through exit survey with 200 insured and uninsured clients, 15 in-depth interviews with health care providers and health insurance managers, and 8 focus group discussions with insured and uninsured community members.

**Results:**

The NHIS promoted access for insured and mobilized revenue for health care providers. Both insured and uninsured were satisfied with care (survey finding). However, increased utilization of health care services by the insured leading to increased workloads for providers influenced their behavior towards the insured. Most of the insured perceived and experienced long waiting times, verbal abuse, not being physically examined and discrimination in favor of the affluent and uninsured. The insured attributed their experience to the fact that they were not making immediate payments for services. A core challenge of the NHIS was a delay in reimbursement which affected the operations of health facilities and hence influenced providers’ behavior as well. Providers preferred clients who would make instant payments for health care services. Few of the uninsured were utilizing health facilities and visit only in critical conditions. This is due to the increased cost of health care services under the NHIS.

**Conclusion:**

The perceived opportunistic behavior of the insured by providers was responsible for the difference in the behavior of providers favoring the uninsured. Besides, the delay in reimbursement also accounted for providers’ negative attitude towards the insured. There is urgent need to address these issues in order to promote confidence in the NHIS, as well as its sustainability for the achievement of universal coverage.

## Background

Health care financing continues to stir debates around the world. Many low and middle income countries especially, keep on exploring different ways of financing their health systems. This is due to the fact that their health systems are chronically under-funded [1]. User fees were initially introduced at the point of service delivery in some of these countries in order to generate revenue for the running of their health systems. In some contexts, the introduction of user fees led to improvement in the quality of health care services [2]. However, the overwhelming evidence suggests that user fees constitute a strong barrier to the utilization of health care services, as well as preventing adherence to long term treatment among poor and vulnerable groups [1,3]. These problems led to yet another debate to look for other alternatives of health care financing.

But prepayment and risk pooling through social health insurance (SHI) and taxation are found to provide protection against some of the undesirable effects of user fees [4]. The international community is therefore paying more attention to SHI as one of the promising financing mechanisms for providing coverage to populations against high health care service costs [5]. SHI is seen as helping to pool health risks, prevent health related impoverishment and improvement in efficiency and quality of health care services [4,6,7]. It also provides access to health care services for the poor and helps mobilize revenue for providers [4]. Nonetheless, the implementation of SHI programmes are challenged in terms of high administrative cost, lack of managerial skills, problems of cost containment and ensuring national coverage [2]. Due to these, there are still few examples of SHI schemes operating at large scale in developing countries [7].

Ghana is among the few African countries that promulgated a National Health Insurance (NHI) law (Act 650). Hitherto, the country had been providing free health care services for her citizens after independence in 1957. This was possible due to the small population size (about 8 million) at the time and a flourishing economy [8]. However, the free health care services could not be sustained because of the economic crisis in the 1970s and early 1980s which adversely affected all sectors of the economy leading to budget cuts on social spending including health and education. Thus, little money was available for the health sector and this led to widespread shortages of essential medicines, supplies and equipment which adversely affected the quality of care in public health facilities [9].

To forestall these problems, cost recovery or user fees (popularly called “cash and carry”) was introduced in the late 1980s in all government facilities. Patients were made to pay for the full cost of medication and care. The argument for the user fees was to generate revenue and to discourage frivolous use of health care services. However, the user fees policy affected the utilization of health care services by Ghanaians. The poor especially, were undertaking self-medication and also reporting late to health facilities for treatment [10,11]. This prompted the need to look for other alternatives of health care financing, which led to the introduction of some Community-based Health Insurance Schemes (CBHIS) in the early 1990s. As at 2003, such CBHIS covered only about 1% of the country’s population (19 million), leaving many Ghanaians uncovered against high health care service costs [12].

### Design and set up of the NHIS

In order to promote universal coverage and equity, the government of Ghana adopted the National Health Insurance Scheme (NHIS) in 2003, which was fully implemented in 2005. The NHIS aims to assure equitable and universal access for all residents of Ghana to an acceptable quality package of essential health care services without OOP payment being required at the point of use [13]. The ultimate goal of the NHIS is the provision of universal health insurance coverage for all Ghanaians, irrespective of their socio-economic background. The NHIS is based on District Mutual Health Insurance Schemes (DMHIS), which operates in all 170 districts of the country. The NHIS covers both the formal and informal sectors of the economy. According to McIntyre et al., the implementation of the NHIS draws experience from the operations of the CBHIS [14]. As at June, 2009, about 67% of the Ghanaian population had subscribed to the NHIS [15].

The NHIS is financed by a national health insurance levy of 2.5% on certain good and services, 2.5% monthly payroll deduction being part of the contribution to the Social Security and National Insurance Trust (SSNIT) for formal sector workers, government budgetary allocation and donor funding. But the formal sector workers will still have to pay a registration fee to a DMHIS for an identity card (ID) to be able to access health care services. Contributions from members of the informal sector are also made to the NHIS with the minimum and maximum premium being GH 7.20 ($8) and 47.70 ($53) respectively [13]. However, the core poor, pregnant women, pensioners, people above the age of 70 and those below 18 years are exempted from premium payment. There is no any other cost sharing or co-payments with the NHIS, except the premium paid.

The benefit package of the NHIS consists of basic health care services, including outpatient consultations, essential drugs, inpatient care and shared accommodation, maternity care (normal and caesarean delivery), eye care, dental care, and emergency care. About 95% of the diseases in Ghana are covered under the NHIS. However, some services classified to be unnecessary or very expensive are on the exclusion list. Among these are; cosmetic surgery, drugs not listed on the NHIS drugs list (including antiretroviral drugs), assisted reproduction, organ transplantation, and private inpatient accommodation.

A fee for service type of provider payment mechanism was used for paying health care providers initially. But this was replaced with the Ghana Diagnostic Related Groupings (GDRGs) in April, 2008. The reason for the replacement was that the fee for each service was found to be low and hence unattractive, especially for the private providers to participate. Providers are encouraged to participate in the NHIS, in order to reduce congestions and delays for clients when seeking health care services. With the fee for service, providers were also required to submit detailed information on all services and charges for claims submissions. This involves a lot of paperwork which providers were not happy with [16].

Hence the GDRGs were introduced to help remedy some of these issues. The tariff covers the full cost of the estimated direct consumables for direct patient care, anesthesia and other investigations. The GDRGs also captures about 80% of the estimated overhead cost for public health facilities, comprising of building and equipment maintenance, housekeeping and utilities [16]. It is expected that the new tariff will generate adequate revenue from the NHIS for providers to cover a significant portion of their cost of operation. But, currently the NHIS is experimenting with capitation in the Ashanti Region of Ghana, to test its feasibility for scaling up, alongside the GDRGs.

Since the inception of the NHIS, many studies have been carried out on the willingness and acceptability of the NHIS, the determinants of enrolment into the NHIS, and the health seeking behavior of insured clients. For instance, a study by Asenso-Okyere et al. found more than 90% of the respondents agreeing to enroll in the NHIS and about 63.6% willing to pay a monthly premium of $3.03 [17]. On determinants of enrollment into the NHIS, it was also shown that individuals from poorer households were less likely to enroll compared with those from rich households [18,19].

However, there is limited knowledge on the influence of the NHIS on the behavior of health care providers. Health care providers form an important segment of health care delivery. Their (providers) behavior plays a significant role in determining whether the goals of a health system can be achieved. The aim of the study was to examine the influence of the NHIS on the behavior of providers towards the insured and uninsured clients. Specifically, the study explored the perceptions of providers about the NHIS, especially the reimbursement process. The perceptions and experiences of insured and uninsured clients, and health insurance managers was also studied and presented.

The study gave an insight and understanding of the issues driving the behavior of health care providers in their treatment of insured and uninsured clients upon the implementation of the NHIS. The information is considered important for Ghana and other countries that are planning to introduce the concept of social health insurance/mandatory health insurance.

## Methods

### Study area and site

The study was carried out in the Upper East Region of Ghana, considered as one of the three poorest regions in the country, with the least number of health workers. Two districts were purposively selected (out of nine districts in the region) for the study, to reflect urban and rural settings. The selected districts were the Bolgatanga municipality and capital town of the region (urban setting) and the Builsa district (rural setting where not many studies have been carried out).

### Study design

The study was cross-sectional and data collection was carried out between December, 2009 and February, 2010. Both qualitative and quantitative methods were used in order to enhance the validity of results through triangulation. Patient exit interviews (survey) were used in the quantitative methods. The main aim of the survey was to gather data on patients’ perceptions and experiences in the two public hospitals in the two districts. Private health facilities were not included for the survey due to time and financial constraints. The qualitative approach used in-depth interviews and focus group discussions (FGDs). The interviews were held with providers, health insurance managers and their claims officers. The purpose of the interviews was to get an insightful data on providers’ perceptions of the NHIS and that of clients. The interviews were also meant to collect insurance managers’ views about the NHIS, providers and clients. The FGDs were conducted among community members. The utilization of the FGDs was also to elicit the perceptions and experiences of community members about the NHIS. Some policy documents were reviewed as well for the study.

### Sampling for the patient exit interviews

A sample size was calculated for the survey in the two districts (Bolgatanga municipality and the Builsa district). Health insurance coverage for the two districts varied, 67% for Bolgatanga [20] and 80% for Builsa [21]. But since the use of a 50% sample size calculation is considered representative, this study used a 50% insurance coverage levels to calculate the sample size for each of the two districts. The essence of the use of the 50% insurance coverage for each district was to obtain an equal sample size to enable comparison between the two groups in the two districts. The calculated sample size for the two districts was 200 (100 for each district and distributed equally between the insured and uninsured).

Systematic random sampling was used to recruit insured clients at the health facilities for the survey. On average 15 participants were recruited a day, using a recruitment interval of 8 patients, starting with the arrival of the first patient. All uninsured clients who presented at the health facilities during recruitment and consented were interviewed. This was done due to their (uninsured) limited use of health care services as a result of the OOP payments. It must be noted that the original intent of the study was to recruit only outpatients; however, it became difficult getting the uninsured clients, especially in the Bolgatanga municipality when the study commenced. It was realized that most of the uninsured visit the facilities as inpatients. Therefore the recruitment was extended to include inpatients in that municipality, to allow for the required sample size to be obtained.

Structured questionnaire were used to collect the required data. These questions were developed in English and translated into the local dialects. A back translation was done for all questions to ensure that their meanings were not lost out as a result of the translation. Questions were based on physical examination at the consultation rooms, waiting times, OOP payments made at the facilities and overall satisfaction with health care provision. Validation of the questionnaire was done through interviews conducted in a public health centre in Navrongo. Two well trained field assistants fluent in the dialects spoken in the chosen districts were responsible for conducting the interviews. Quality was monitored by the investigators observing on average, two interviews a day.

### In-depth interviews

Fifteen (15) in-depth interviews were conducted with providers and health insurance managers in the two districts (8 interviews in Bolgatanga and 7 in Builsa). Semi-structured interview guide was used for conducting the interviews, which was in English because all the participants could speak English fluently. The interviews covered the reimbursement process, the response of providers to the reimbursement process, providers’ treatment of insured clients and challenges facing the NHIS. Piloting of the interview guide was done with providers in the Health Centre in Navrongo. The interviews were recorded with a digital voice recorder. The recordings were listened to and transcribed for the data analysis. Field notes were also taken alongside as a complement. The investigators carried out all the in-depth interviews.

### Focus group discussions (FGDs)

**Figure 1:**
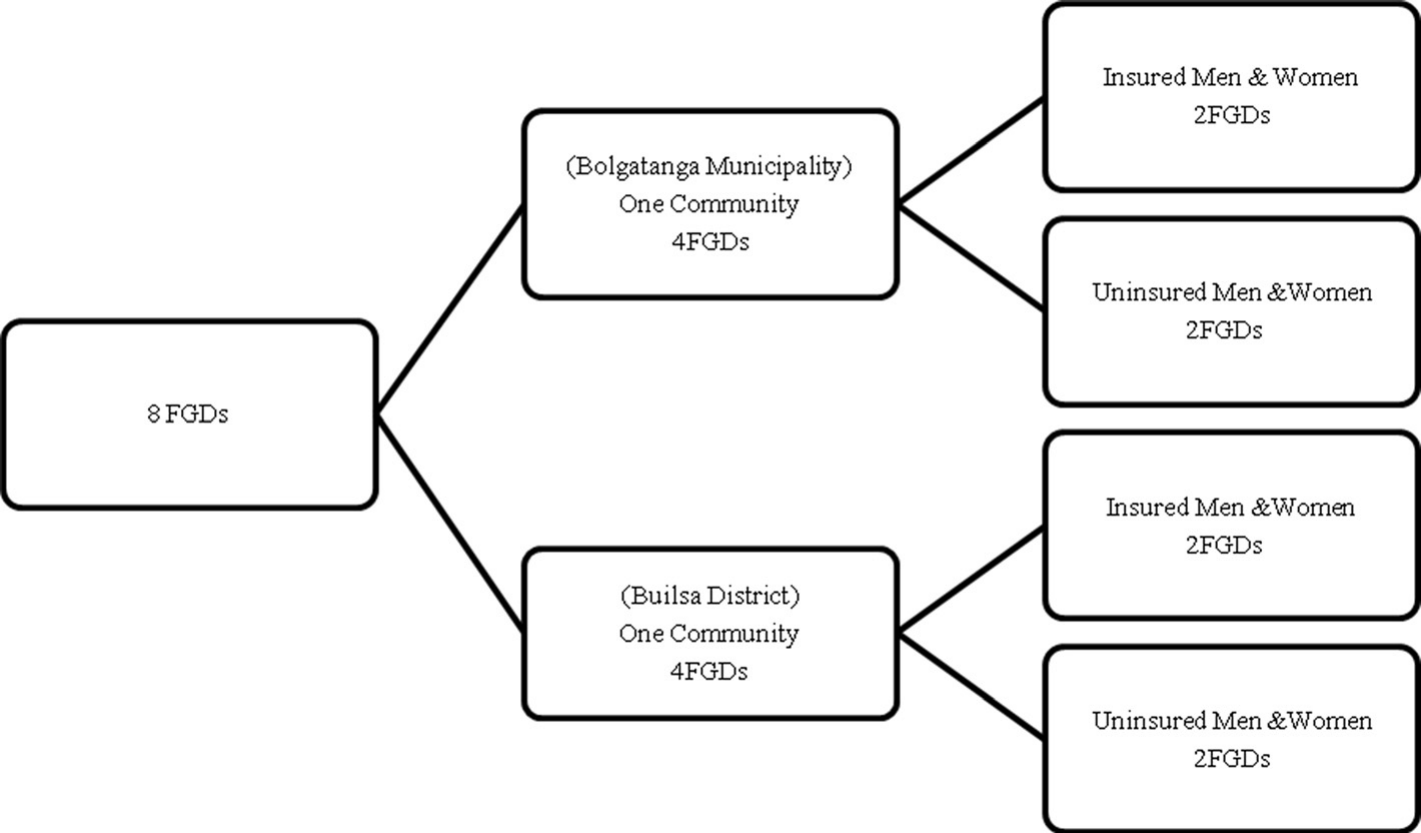
Design for the focus group discussions.

Eight (8) FGDs were held with community members in the two districts. The discussions were conducted with both insured and uninsured men and women between the ages of 18 and 60 years (both productive and reproductive ages). Figure 1 is a diagrammatic representation of the design for the discussions.

From Figure 1, one community each from the two districts was selected for the FGDs. This selection was done according to the community’s location from the health facility because the location of a community to a facility can determine the health seeking behavior of its residents. A community that extended out from the facility up to about 8 kilometers (kms) was the criteria for its inclusion in the study. A community that extends about 8kms from a facility includes households that are nearer to the facility (about 4kms within) and also households that are far from the facility (about 4kms further). Zaare in Bolgatanga and Tankonsa in Builsa were the communities selected and used for the discussions. The selection of the participants for the FGDs was also done taking into account their residential location to the facilities in the two districts. The study brought together participants who lived nearer (about 4kms within) and those who lived further (about 4kms further) from the facilities for the discussions. Each discussion group had a membership of 8 to 12 members.

An interview guide was developed in English and translated into the local dialects by experts as well. The guide contained issues on the perceptions and experiences of community members about the NHIS and that of providers’ behavior. A pilot test of the interview guide was done in the Pungu community in Navrongo. The discussions were recorded and transcribed into English. All transcriptions were done verbatim to reduce errors. Field notes were also taken, to complement the transcribed notes. The field assistants conducted the FGDs which were supervised by the investigators.

### Data analysis

The data from the survey was entered into EpiData software (3.1) and later transferred to STATA, (version 10.0) for analysis. The data was entered twice as a form of validation check, to help detect errors and inconsistencies. The identification of outliers and other inconsistencies among the variables was done through the running of frequencies and cross tabulations. Chi-square test was used to test for differences in groups and *t*-test for differences in means. The in-depth interviews and FGDs were recorded and transcribed. This data was analysed manually using themes. The investigators read through all the transcripts exhaustively and coded them. The codes were matched and generated into common themes and sub-themes for the write-up.

### Ethical considerations

Ethical approval was granted for the study by the Ghana Health Service Ethical Review Board, Ghana. Permission for the research was also obtained from hospital management and all participants gave informed written consent to be interviewed or surveyed.

## Results

This section comprises of the results for both the survey and the qualitative study. A total of 200 respondents were recruited for the survey comprising of 100 participants each in two public facilities in the Bolgatanga and Builsa districts. The 100 respondents in each district were stratified equally into insured and uninsured positions. There were also 15 in-depth interviews and 8 focus group discussions held for the qualitative study.

### Characteristics of respondents

Table 1 is a composition of the characteristics of respondents for the survey. About 51% of the respondents were in the age range of 21–60 years who were uninsured while 49% were in the 0–20 age range and were insured.

**Table 1:** Characteristics of respondents

**Characteristics**	**Bolgatanga municipality**				**Builsa district**				**Total**			
	**Insured**		**Uninsured**		**Insured**		**Uninsured**		**Insured**		**Uninsured**	
** Age (years)**	**Freq (50)**	**%**	**Freq (50)**	**%**	**Freq (50)**	**%**	**Freq (50)**	**%**	**Freq (100)**	**%**	**Freq (100)**	**%**
0-20	20	40	21	42	29	58	24	48	49	49	45	45
21-60	29	58	28	56	16	32	23	46	45	45	51	51
61+	1	2	1	2	5	10	3	6	6	6	4	4
**Sex**												
Male	23	46	24	48	20	40	21	42	43	43	45	45
Female	27	54	26	52	30	60	29	58	57	57	55	55
**Type of patient**												
Inpatient	15	30	31	62	-	-	-	-	15	15	31	31
Outpatient	35	70	19	38	50	100	50	100	85	85	69	69

Females were more than the male respondents, with 57% being insured and 55% being uninsured. Outpatients who were insured were 85% while the uninsured were 69%. Insured and uninsured inpatient respondents were 15% and 31% respectively (Note: the inpatients came from the Bolgatanga municipality only). Demographic characteristics of respondents for the in-depth interviews and focus group discussions were not documented.

### Diagnosis

Causes of outpatient attendance and hospitalizations are presented in Table 2. Malaria tops the list and accounts for 55% of the insured and 60% of the uninsured attendance. In the Bolgatanga municipality, malaria was responsible for 33.3% insured and 67.8% uninsured inpatient hospitalization. It also caused 60% and 58% insured and uninsured outpatients attendance in the Builsa district.

**Table 2:** Diagnosis

**Diagnosis**	**Bolgatanga municipality**								**Builsa district**				**Total**			
	**Insured**				**Uninsured**				**Insured**		**Uninsured**		**Insured**		**Uninsured**	
	**Inpatients**		**Outpatients**		**Inpatients**		**Outpatients**		**Outpatients**		**Outpatients**					
	**Freq**	**%**	**Freq**	**%**	**Freq**	**%**	**Freq**	**%**	**Freq**	**%**	**Freq**	**%**	**Freq**	**%**	**Freq**	**%**
Malaria	5	33.3	20	57.1	21	67.8	10	52.6	30	60	29	58	55	55	60	60
URI	1	6.7	4	11.4	1	3.2	3	15.8	5	10	6	12	10	10	10	10
Anemia	2	13.3	3	8.6	-	-	2	10.5	4	8	5	10	9	9	7	7
Others	7	46.7	8	22.9	9	29	4	21.1	11	22	10	20	26	26	23	23
**Total**	**15**	**100**	**35**	**100**	**31**	**100**	**19**	**100**	**50**	**100**	**50**	**100**	**100**	**100**	**100**	**100**

### Various views about the NHIS

The qualitative aspect of the study sought the views of the insured, uninsured, health care providers and health insurance managers about the NHIS in both districts. Below is a compilation of these views.

### Views of insured clients

The insured discussants all agreed that the NHIS is very useful. It made access to health care services very easy. This was due to the fact that one was not required to pay for services at the point of consumption:

It is good because we in this community are farmers and poor, when you are sick, at once you cannot sell a fowl to be able to go to the hospital. But with the insurance, we are safe now (FGD, insured men-Builsa).

But the insured clients were not happy with high premium payment for registration, the delay in processing the insurance ID cards after registration, and the yearly renewal of the ID cards. Besides the perceived limited benefit package of the NHIS and the unreliable nature of the insurance agents were other issues the insured were not pleased with. The insurance agents were accused of charging unofficial fees and also causing delays in the processing of the insurance ID cards.

### Views of uninsured clients

The uninsured clients also recognized the usefulness of the NHIS. They reported that they had seen insured clients accessing health care services without any payment, even with free feeding for those hospitalized:

The insurance helps in the sense that when you are admitted in the hospital it is free, you get everything for free; even the food and the bed free (FGD, uninsured men-Bolgatanga).

The uninsured were of the view that the premium paid was high and hence a barrier for them to register with the NHIS:

Most of us in this community are farmers, with our incomes being unstable. It is very difficult getting that amount of money to join the insurance (FGD, uninsured women-Builsa).

The delay in obtaining the insurance ID cards and their yearly renewal were other issues that the uninsured complained about concerning the NHIS. The above issues therefore do not motivate them (uninsured) to subscribe to the NHIS:

We have heard people complaining of delays and the yearly renewal of their insurance ID cards which is a bad thing. This doesn’t encourage our registration (FGD, uninsured men-Builsa).

### Views of health care providers

Health care providers in the two districts indicated that the NHIS enabled providers to get funds in bulk to carry out their operations and to undertake minor infrastructural developments in the facilities. It had also made health care services accessible to the insured without any payment at the point of consumption. This was reflected in the high attendance by the insured. They attested to a phenomenal increase in attendance compared to the period when the NHIS was not in operation:

The NHIS assisted in health care delivery. The insured are able to access health services all the time without any instant payment (In-depth interview, hospital accountant-Bolgatanga).

But providers perceived that the introduction of the NHIS had led to service abuse by the insured. The insured frequent the facilities with minor ailments and even attend to collect drugs for their uninsured relatives and friends. Some insured clients even offer their insurance ID cards to the uninsured for a fee to use to access health care:

*Some people can come to the hospital ten times in a week. There is an abuse by the insured (In-depth interview, hospital accountant-Bolgatanga)*.

The high attendance and perceived service abuse by the insured had led to an increased workload for providers. Providers experience long working hours with little or no break times. However, providers were not motivated enough by the NHIS and government to compensate for the heavy workload experienced.

A major challenge disclosed by providers was a delay in reimbursement. Providers were not paid for over six (6) months in both districts:

For almost six (6) months, the insurance has not paid the hospital (In-depth interview, hospital administrator-Builsa).

But the NHI Act (650) stipulates that providers should be reimbursed four weeks following the month for which claims were submitted. This was not the case and hence contravening the NHI Act (650).

The main reason for the delay in payment was the inability of the National Health Insurance Authority (NHIA) to provide funds for payment. The NHIA seems to be overwhelmed with the amount of claims submitted by the various DMHIS for payment. This seems to be a result of the introduction of the new GDRG which had higher rates for payment than the previously fee for service charge.

But other reasons that could result in delays in payment included inadequate and incompetent staff in the facilities who were responsible for the submission of claims to the DMHIS. For instance, some staff lacked knowledge of the computer software used for submitting claims to the DMHIS. Contentious claims between the facilities and the DMHIS could sometimes result in delays as well.

Due to the delay in reimbursement, providers were unable to procure drug and non-drug supplies for the smooth operations of the facilities:

The delay affects our work, because if you don’t have the logistics to work with...tell me, assuming that you have run out of drugs, they (NHIA) have not paid you, where will you get the money to buy the drugs? (In-depth interview, hospital administrator-Builsa).

The delay in payment had made providers resort to the issuance of prescription forms for insured clients to buy drugs out of the facilities.

Another consequence of the delay in reimbursement was the fact that it had made some providers prefer clients who would make OOP payments for services to those with the NHIS cards:

Some facilities in the South of the country have turned away people who are insured. It is true that some facilities and pharmacies would prefer people who will pay in cash to those with insurance, due to the delay in reimbursement (In-depth interview, hospital accountant-Bolgatanga).

These OOP payments from the uninsured assist providers to run the facilities while they wait for the main payments from the NHIS.

In addition, providers were handicapped in the payment of their casual employees. For example, cleaners and security men whose names were not on government’s payroll are usually paid from internally generated funds mobilized by the facilities.

### Views of health insurance managers

Insurance managers considered the NHIS to be one of the best social interventions that enabled clients to have access to health care services as and when needed:

As at now, insured members are accessing health services without the problem of having to pay at the point of use (In-depth interview, insurance manager-Builsa).

Insured members do not wait for their health problems to deteriorate before they seek health care. A number of operations had been carried out especially for people with hernia. A health insurance manager remarked:

Our region is hard hit with hernia problems and the NHIS has helped to reduce this problem. A substantial number of operations has been done (In-depth interview, insurance manager-Bolgatanga).

### Perception of waiting times at the facilities

Insured and uninsured clients’ views of waiting times in the facilities have been captured in Table 3. While no difference was observed for inpatients of insured and uninsured status in the Bolgatanga municipality (p-value = 0.345), there was a difference between outpatients who are insured and those who are uninsured (p-value = 0.003). About 76% of outpatients who are insured compared to about 63.16% uninsured perceived the waiting times to be too long. In the Builsa district, the insured (68%) regarded waiting times to be too long (p-value = 0.000) as well. Further, a difference (p-value = 0.001) had been found for the combined result from the two districts between the insured and uninsured. About 59% of insured respondents viewed the waiting times to be too long compared to only 35% of the uninsured.

**Table 3:** Perception of waiting times at the facilities

**Waiting times?**	**Bolgatanga municipality**										**Builsa district**					**Total**				
	**Inpatients**					**Outpatients**					**Outpatients**									
	**Insured**		**Uninsured**		P-value*	**Insured**		**Uninsured**		P-value*	**Insured**		**Unisured**		P-value*	**Insured**		**Uninsured**		P-value*
	**Freq**	**%**	**Freq**	**%**		**Freq**	**%**	**Freq**	**%**		**Freq**	**%**	**Freq**	**%**		**Freq**	**%**	**Freq**	**%**	
Too long	6	24	7	36.84	**0.345**	19	76	12	63.16	**0.003**	34	68	16	32	**0.000**	59	59	35	35	**0.001**
Ok	9	36	24	77.42		16	64	7	16.22		16	32	34	68		41	41	65	65	
Total	**15**	**30**	**31**	**62**		**35**	**62**	**19**	**38**		**50**	**100**	**50**	**100**		**100**	**100**	**100**	**100**	

* Chi-square test to compare proportions for insured and uninsured respondents.

This finding was supported by the qualitative study. The results showed that the insured complained of long waiting times. This was what an insured client said:

We come to the hospital early in the morning and leave only in the evening for home. We spend the whole day in order to get treatment (FGD, insured men-Bolgatanga).

The health insurance managers and providers also agreed that waiting times for the insured was longer than for the uninsured due to the documentation process that the insured need to undergo.

### Views on physical examination of clients by health care providers

Table 4 consists of the responses on whether clients had been physically examined in the consultation room by providers. Except for the insured and uninsured outpatients in the Bolgatanga municipality (p-value = 0.263), the rest of the results indicated a significant difference for the two groups. More of the uninsured reported having been physically examined than the insured. About 94% of the uninsured outpatients in the Builsa district responded “yes” to being physically examined by providers compared to 78% of the insured outpatients (p-value = 0.021). This pattern was also observed for the total responses between the two groups, where 81% of the uninsured reported having been physically examined compared to 68% of the insured respondents (p-value = 0.035).

**Table 4:** Views on physical examination of clients by health care providers

**Physically examined?**	**Bolgatanga municipality**										**Builsa district**					**Total**				
	**Inpatients**					**Outpatients**					**Outpatients**									
	**Insured**		**Uninsured**		P-value*	**Insured**		**Uninsured**		P-value*	**Insured**		**Uninsured**		P-value*	**Insured**		**Uninsured**		P-value*
	**Freq**	**%**	**Freq**	**%**		**Freq**	**%**	**Freq**	**%**		**Freq**	**%**	**Freq**	**%**		**Freq**	**%**	**Freq**	**%**	
Yes	12	41.38	23	67.65	**0.040**	17	58.62	11	32.35	**0.263**	39	78	47	94	**0.021**	68	68	81	81	**0.035**
No	3	14.29	8	50		18	85.71	8	50		11	22	3	6		32	32	19	19	
**Total**	**15**	**30**	**31**	**62**		**35**	**70**	**19**	**38**		**50**	**100**	**50**	**100**		**100**	**100**	**100**	**100**	

*Chi-square test for comparing proportions for insured and uninsured respondents.

The point was buttressed by the qualitative study as well. Some insured discussants were not physically examined by providers before prescribing drugs for them. This was seen in both districts. An insured client said:

..after my complaints, the doctor just wrote some drugs for me, he did not touch any part of my body (FGD, insured women-Builsa).

### Direct payments for health care services

The uninsured and some insured patients made direct OOP payments at the facilities for drugs and services. Table 5 is a detail of these payments. The mean OOP payments for insured (8 in number) and uninsured respondents (50 in number) in Bolgatanga was GHÂ¢ 7.625 (USD 5.37) and GHÂ¢ 23.74 (USD 16.63) respectively (regardless of patient type). Also, the mean OOP payments for inpatients (35 in number) and outpatients (23 in number) in the municipality was GHÂ¢ 24.91429 (USD* 17.545) and GHÂ¢21.24685 (USD* 14.962) in the given order (irrespective of insurance status). Uninsured outpatient respondents in Builsa district made a mean OOP payments of GHÂ¢23.5 (USD 17.96) for health care services. But insured outpatient respondents made no OOP payments in the district. The overall result from the two districts showed a mean OOP payment of GHÂ¢23.62 (USD*16.63) for the uninsured (100 in number) and GHÂ¢7.625 (USD*5.37) for the insured (8 in number).

**Table 5:** Direct payments for health care services

	**Insurance status**	**Obs**	**Mean (GH₵)**	**Std dev (GH₵)**	**Insurance status not considered**	**Obs**	**Mean (GH₵)**	**Std dev (GH₵)**
**Bolgatanga municipality**	**Insured**	8	7.625	9.164177	**Inpatient**	35	24.91429	12.97593
	**Uninsured**	50	23.74	11.34579	**Outpatient**	23	16.34783	9.398301
**Builsa district**	**Insured**	-	-	-	**Inpatient**	-	-	-
	**Uninsured**	50	23.5	14.16124	**Outpatient**	50	23.5	14.16124
**Total**	**Insured**	8	7.625	9.164177	**Inpatient**	35	24.91429	12.97593
	**Uninsured**	100	23.62	12.76658	**Outpatient**	73	21.24658	13.21592

*Exchange rate: GHÂ¢1 .42 = USD 1 (15/03/2010).

From the qualitative study, the introduction of the NHIS had made the cost of services to rise. The new increased charges implemented by the NHIS for payment to providers for their diagnosis and treatment were the same charges that the uninsured would have to pay when they visit the facilities:

These days the cost of seeking health care services is very expensive. The worst thing is when you are on admission, they may charge you between GHÂ¢ 300 and GHÂ¢ 400 (US $ 263.15 and US $ 350.88) for just 2 or 3 days. It wasn’t like that (FGD, uninsured women-Bolgatanga).

The high service charge had become a challenge for the uninsured when accessing services. The result is low utilization rates. Providers revealed that only few of the uninsured were visiting the facilities and mostly in critical conditions:

We have realized that with the uninsured, unless they are seriously ill they don’t report early to the facilities. Some of them just come to die (In-depth interview, medical doctor-Builsa).

### Actual time spent by clients at the facilities

Time spent at the facilities by respondents is shown in Table 6. The overall result from the two districts showed a difference (p-value = 0.0274) in time spent by the insured and uninsured outpatient respondents. The insured outpatients spent a mean time of 236.8 minutes while the uninsured outpatient respondents spent a mean time of 203.0 minutes seeking health care services. A similar observation was made in the Builsa district, where the insured outpatient respondents spent a significant mean time of 238.1 minutes seeking health care likened to a mean time of 199.6 minutes spent by the uninsured (p-value = 0.0096). No mean time difference was observed for insured and uninsured inpatients (p-value = 0.0890) and outpatients (p-value = 0.5238) in the Bolgatanga municipality.

**Table 6:** Actual time spent by clients at the facilities

	**Inpatient (days)**					**Outpatient (minutes)**			
		**Obs**	**Mean**	**Std dev**	**P-value**	**Obs**	**Mean**	**Std dev**	**P-value**
**Bolgatanga municipality**	**Insured**	15	2.533333	1.302013	**0.0890**	35	234.8857	122.8059	**0.5238**
	**Uninsured**	31	3.677419	2.371878		19	212.1053	127.7684	
**Builsa district**	**Insured**	-	-	-	**-**	50	238.1	47.92607	**0.0096**
	**Uninsured**	-	-	-		50	199.56	91.35894	
**Total**	**Insured**	15	2.533333	1.302013	**0.0890**	85	236.7765	86.2944	**0.0274**
	**Uninsured**	31	3.677419	2.371878		69	203.0145	101.8208	

*t-test to compare mean time spent by insured and uninsured respondents.

The interviews with the health insurance managers collaborated the finding of the survey by confirming that the insured experience a lot of delays when seeking health care:

*We’ve received numerous complains of delays by our clients at the facilities. Some insured clients spend a whole day seeking health care in the facilities (In-depth interview*, *insurance manager-Bolgatanga).*

This situation does not encourage the insured to attend the facilities when ill as they use to when the NHIS was initially introduced.

### Overall satisfaction with health care provision

Table 7 shows the overall level of satisfaction of respondents with health care service provision in the two districts and facilities. In all, there was no difference (p-value = 0.177) in response with regards to satisfaction with health care service provision between the insured and the uninsured. About 76% of the insured and 82% of the uninsured were satisfied with the health care provided them. The same pattern had been observed between inpatients of insured and uninsured positions (p-value = 0.734) as well as outpatients who are insured and uninsured (p-value = 0.240) in the Bolgatanga municipality. Equally, the Builsa district saw no difference in response for the insured and uninsured outpatients (p-value of 0.292).

**Table 7:** Overall satisfaction with health care provision

**Satisfaction with health care?**	**Bolgatanga municipality**										**Builsa district**					**Total**				
	**Inpatients**					**Outpatients**					**Outpatients**									
	**Insured**		**Uninsured**		P-value*	**Insured**		**Uninsured**		P-value*	**Insured**		**Uninsured**		P-value*	**Insured**		**Uninsured**		P-value*
	**Freq**	**%**	**Freq**	**%**		**Freq**	**%**	**Freq**	**%**		**Freq**	**%**	**Freq**	**%**		**Freq**	**%**	**Freq**	**%**	
Satisfied	10	31.25	23	67.65	**0.734**	22	68.75	11	32.35	**0.240**	44	88	48	96	**0.292**	76	76	82	82	**0.177**
A little satisfied	4	25	8	50		12	75	8	50		5	10	2	4		21	21	18	18	
Dissatisfied	1	50	-	-		1	50	-	-		1	2	0	0		3	3	0	0	
Total	**15**	**30**	**31**	**62**		**35**	**70**	**19**	**38**		**50**	**100**	**50**	**100**		**100**	**100**	**100**	**100**	

*Chi-square test to compare proportions for insured and uninsured respondents.

However, the qualitative study revealed that the insured were not pleased with the service provision. Most insured clients in both districts perceived that providers discriminated against them by causing delays for them when they come for their hospital folders at the records unit. Providers also prescribe low quality drugs for them, issue prescription forms for them to buy drugs out of the facilities, and sometimes verbally attacking them for no apparent reason:

Some of the staff are too discriminatory. When you come without insurance, very quickly they will attend to you (FGD, insured men-Builsa).

It was also reported by the insured that providers tend to give preferential treatment to the rich, who were well dressed and attended the facilities in cars. They (insured) contended that the rich were attended to quickly than the poor, but discussants did not indicate whether these rich people were insured or uninsured clients:

They have their own people who are well dressed and good looking, but not poor people like me (FGD, insured women-Builsa).

The insured therefore concluded that it was because they were not making instant payment for health care services, they were being discriminated against by the providers.

## Discussion

The study found that the NHIS is working, promoting access for the insured and mobilizing revenue for providers. Both the insured and uninsured were satisfied with care provided (survey findings). However, most insured clients had reported long waiting times, verbal abuse, not being physically examined and discrimination. Providers perceived that the insured were abusing their services and generating lots of workload for them. The uninsured were found not to be utilizing the facilities.

Firstly, the finding that utilization of health care services had increased under the NHIS, are confirmed by other studies. For instance, both baseline and endline studies on the NHIS saw an increase in utilization of health care services from 37% in 2004 to 70% in 2008 in Ghana [22]. Similarly, the Ministry of Health (Ghana) reported that the use of outpatient and inpatient services under the NHIS almost doubled between 2005 and September 2007 [23]. A recent study in the Volta Region of Ghana, also found the NHIS to have positively affected health seeking behavior and the consumption of health care services [24]. Elsewhere in Burkina Faso, Gnawali et al. reported higher utilization rates (about 40% higher) for outpatient services under the Community-based Health Insurance Scheme [25]. Our finding on increased utilization could be explained by some unmet health care needs prior to the introduction of the NHIS, especially when the “cash and carry” was in operation. It could also be explained by an abuse on the part of insured clients as perceived by providers.

On the issue of the delay in reimbursement, various news items had been put up by Ghanaweb.com in Ghana showing some providers refusing to offer services to some insured clients unless they were ready to make instant payments. For instance, it was reported by the Ghana News Agency that the Ho Municipal Hospital (in the Volta region, Ghana) had turned away insured clients unless they were ready to pay for the services [26].

The delay in reimbursement might be one of the underlying reasons for the differences in the behavior of providers towards the insured and uninsured. This will require action by policy makers to address the issue in order to promote the sustainability of the NHIS and to make possible the attainment of universal coverage.

Secondly, the combined result for the two districts and that of Builsa revealed that the insured perceived waiting times to be long. The same trend was observed when the actual time spent at the facilities was determined for both insured and uninsured. Insured outpatients spent more time in the facilities than the uninsured. The finding is in line with what Bruce and colleagues identified, that clients making OOP payments for health care services had shorter waiting times than their counterparts who were carrying health insurance cards [27]. In Burkina Faso too, De Allegri et al. found insured respondents complaining of long waiting times when they access health care services [28]. Our finding showed a difference in waiting times between the two districts which could be explained by the fact there was more medical staff in Bolgatanga than in Builsa. For instance, the Bolgatanga Regional hospital had 5 medical doctors, while Builsa had just one. But providers explained that the differences in waiting times by the insured and uninsured was due to the processes that the insured goes through in terms of documentation. Given their (insured) high attendance rates, waiting times were likely to be high for them. It would therefore be appropriate if the NHIS or policy makers are able to put measures in place to reduce the documentation process for the insured and help bring down their waiting times.

Thirdly, the findings from the survey and FGDs proved that more of the uninsured than the insured reported having been physically examined by providers. The finding runs contrary to that of Bassili et al. Their study in Egypt, found insured clients to have had a significantly higher frequency of physical examination, laboratory investigations and diabetes education compared to their uninsured colleagues [29]. However, our finding could be explained by the high attendance of the insured, creating more pressure on providers, thus making physical examination a time-consuming exercise. With this finding, it is required that a continuous monitoring of the provision of health care services is undertaken to ensure that services rendered to insured clients are of the required standard.

Fifthly, the overall mean for direct OOP payments for drugs and services for the uninsured was GHÂ¢23.62 (USD16.63). Proportionally, this payment would be 1.2% to the average annual household expenditure of GHÂ¢ 1,918.00 (USD 1,350) in Ghana [30]. Indirect costs were not included, but are known to be quite significant when seeking health care services in Ghana [31]. A direct cost of GHÂ¢23.62 (USD16.63) for one episode of illness could therefore be considered high. Besides, the FGDs showed that most of the uninsured were the poorest and hence that payment would have a significant negative impact on them. In fact, the literature on health insurance in Africa have shown that the poorest were not represented among the insured [28,32,33], and could adversely be affected by direct OOP payments. Special efforts are required to include this class of people in the NHIS. But the insured who made OOP payments were for services or drugs not covered by the benefit package of the NHIS.

Lastly, the survey showed that the insured and uninsured were satisfied with health care provision under the NHIS. This conforms to a study exploring the level of satisfaction among insured and uninsured clients under two CBHIS in India. It showed no significant difference in the level of satisfaction for the insured and uninsured for the two schemes [34]. However, the FGDs with community members revealed that the insured were less satisfied with the provision of health care than the uninsured. This is also supported by Bruce et al. where the uninsured were more satisfied with the quality of care than the insured [28]. The finding underscores the fact that some consumers of health care services, especially the insured are not happy with the services rendered them in the era of the NHIS. This requires further investigation as to why there is dissatisfaction with service delivery which this study did not address. Once more, the NHIA should endeavor to provide continuous monitoring and evaluation of the services provided by providers to ensure confidence and satisfaction.

### Limitations of the study

The study was conducted in only two of Ghana’s 170 districts, which may limit generalizing the results. In addition, the study was carried out in only two public health facilities, despite the visible existence of the private sector. About 1,277 private health facilities have been accredited, providing about 10% of health care services in Ghana [35]. This therefore affects the representativeness of the facilities surveyed. A study in two public health facilities would not be enough to represent both the public and private health facilities.

Also, the inclusion of inpatients in the Bolgatanga municipality to make up for the required number of respondent introduces a weakness in the sampling strategy for the survey. This limits the consistency of the analysis (as planned initially to compare insured and uninsured outpatients). Besides, it does not permit comparison to be made between the two districts, as it is inappropriate to compare the results of outpatients and inpatients as they receive different types of health care services. Lastly, it restricts the conclusions that can be made about the results from the two districts. Hence the interpretation of the results should be made with caution.

In addition, non-medical costs such as cost of meals, transportation, etc., were not considered by the study and hence the cost captured here would be an under-estimation of the total OOP payments.

### Further research

There is the need for further research to cover a lot more of the districts and in more public and private facilities to help determine the influence of the NHIS on the behavior of providers. It would particularly be interesting to study the behavior of providers in the private sector since they are profit-oriented. Further research should also be carried out to determine if non-medical costs in terms of transportation, meals, etc., affect health seeking among the population, apart from removing payments at the point of service use.

### Policy recommendations

The immediate policy required would be on the issue of the delay in reimbursement by the NHIS. There is an urgent need for action to streamline the reimbursement procedure in order to maintain providers’ confidence in the NHIS. Central government and the management of the NHIA should search for an effective and permanent way of addressing the issue, if not the success and sustainability of the NHIS would be affected.

Another issue is the high cost of health care services in the era of the NHIS. The introduction of the new tariff (Ghana Diagnostic Related Groupings) in April 2008 had led to an increase in the cost of health care services [16]. This therefore affected the utilization of services by the uninsured.

Other issues included high premium payment leading to the exclusion of the poorest, long waiting periods for the issuance of insurance ID cards, required annual renewal of the ID cards, perceived limited benefit package of the NHIS, and issuance of prescription forms for clients to buy drugs out of the facilities. The unacceptable behavior of some insurance agents contracted by the NHIS to register members was another challenge. The above problems require action to ensure the success of the NHIS in providing health care services to the population, particularly mapping out strategies to include the very poor, in order to attain universal coverage. On the part of the facilities, more professional staff are required. The staff should also be motivated to carry out their duties efficiently due to the heavy workloads.

## Conclusion

The study set out to examine the behavior of providers under the NHIS. This was achieved by assessing the views of providers, insurance managers, and insured and uninsured clients. The perceived opportunistic behavior of the insured by providers was responsible for the difference in the behavior of providers favoring the uninsured. Besides, the delay in reimbursement also accounted for providers’ negative attitude towards the insured.

The NHIS was seen by all participants of the study to be beneficial. It led to an increase in the utilization of health care services for the insured and mobilized health resources for facilities. The insured and uninsured were satisfied with the care given them, according to the survey. However, most insured clients reported verbal abuse, long waiting times, not being physically examined and discrimination in favor of the uninsured and the rich. Providers also think that the insured were abusing their services by frequenting the facilities, and sometimes faking illness to collect drugs for their uninsured relatives. This had affected significantly the behavior of providers towards the insured.

One of the biggest challenges was the delay in reimbursement. Managers and providers agreed that the NHIA had not reimbursed providers for almost six (6) months. As a result, providers were not able to purchase drugs and non-drug supplies and hence were prescribing drugs for the insured especially, to purchase outside the facilities. The delay also affected providers’ ability to pay their casual employees who were not on government’s payroll. This again, influenced the behavior of providers where some of them preferred clients who would make instant payments for care. There is urgent need to address these issues in order to promote confidence in the NHIS, as well as its sustainability for the achievement of universal health insurance coverage.

## Abbreviations

CBHIS: Community-based Health Insurance Schemes; DMHIS: District mutual health insurance schemes; FGDs: Focus group discussions; GDRGs: Ghana diagnostic related groupings; NHI: National health insurance; NHIA: National health insurance authority; NHIS: National health insurance scheme; OOP: Out-of-pocket; SHI: Social health insurance; SSNIT: Social security and national insurance trust.

## Competing interest

The authors declare that they have no competing interests.

## Authors' contributions

PAD conceived and designed the study, and drafted the manuscript. ASL participated in designing and coordinating the study, and also revised the manuscript. All authors read and approved the final manuscript.
